# Effect of vitamin D status and vitamin D supplementation on immune function and prevention of acute respiratory tract infections in dark-skinned individuals: a systematic review and meta-analysis

**DOI:** 10.1017/S1368980024001861

**Published:** 2024-10-28

**Authors:** Abigail R Bournot, Andrea L Darling, Ian D Givens, Julie A Lovegrove, Susan A Lanham-New, Kathryn H Hart

**Affiliations:** 1 Department of Nutrition, Food & Exercise Sciences, School of Biosciences and Medicine, Faculty of Health and Medical Sciences, University of Surrey, Guildford, UK; 2 Institute for Food, Nutrition and Health, University of Reading, Reading, UK; 3 Hugh Sinclair Unit of Human Nutrition, University of Reading, Reading, UK

**Keywords:** Vitamin D, 25(OH)D, Ethnic group, Race/ethnicity, Immune function, Respiratory tract infection

## Abstract

**Objective:**

This systematic review and meta-analysis examined the evidence for a potential relationship between vitamin D status and vitamin D supplementation on immune function biomarkers and prevention of acute respiratory tract infections (ARTI) in dark-skinned individuals.

**Design:**

Six databases were searched (inception to December 2021) for randomised controlled trials (RCT) and observational studies. A narrative synthesis and random-effects meta-analysis were used to synthesise the findings.

**Setting:**

Not applicable.

**Participants:**

Ethnic groups other than white, with or without a white comparator.

**Results:**

After duplicates were removed, 2077 articles were identified for screening. A total of eighteen studies (*n* 36 707), including seven RCT and 11 observational studies, met the inclusion criteria, and three RCT (*n* 5778) provided sufficient data of high enough quality to be included in a meta-analysis. An inverse association between vitamin D status and at least one inflammatory biomarker in black adults was found in three studies, and vitamin D status was inversely associated with ARTI incidence in black and Indigenous groups in two studies. There was no significant effect of vitamin D supplementation on differences in ARTI incidence in ethnic minority groups (OR, 1·40; 95 % CI: 0·70, 2·79; *P* = 0·34), nor African American (OR, 1·77; 95 % CI: 0·51, 6·19; *P* = 0·37) or Asian/Pacific (OR, 1·08; 95 % CI: 0·77, 2·68; *P* = 0·66) subgroups.

**Conclusions:**

There is a lack of conclusive evidence supporting an association between vitamin D status and immune function or ARTI incidence in dark-skinned individuals. Further RCT in diverse ethnic populations are urgently needed.

Vitamin D deficiency (defined as serum 25-hydroxyvitamin D (25(OH)D) concentrations <25 or <30 nmol/l^(1,2)^) is a global health concern, with dark-skinned ethnic groups found to exhibit a higher prevalence compared to populations of white European ancestry^(3,4)^. In the US, data from the 2007 to 2018 National Health and Nutrition Examination Survey showed that the prevalence of vitamin D deficiency (<25 nmol/l) was highest in non-Hispanic black populations (56·6 %) compared to non-Hispanic white (26·4 %), Other (10·1 %) and Mexican American populations (6·9 %)^(5)^. In the UK, an analysis of data from the UK Biobank cohort reported the prevalence of vitamin D deficiency (<25 nmol/l) to be highest in South Asian populations at 61·4 %, followed by 34·5 % in African populations, and 11·5 % in white populations^(4)^. Additionally, dark-skinned ethnic groups have been disproportionately affected by pandemics caused by respiratory viruses, such as the 2009 Influenza A (H1N1) pandemic^(6)^ and coronavirus disease 2019 (COVID-19) pandemic^(7,8)^. In the COVID-19 pandemic, these disparities were not fully explained by sociodemographic characteristics and comorbidities, suggesting alternative factors may be involved^(7)^. Lower vitamin D status in these populations has been suggested as a contributing factor, yet the evidence linking vitamin D and acute respiratory tract infections (ARTI) remains limited^(9)^.

While existing guidelines for vitamin D supplementation have been largely informed by evidence on musculoskeletal health requirements^(10)^, recent evidence suggests that vitamin D plays a role in both immune modulation and infectious disease^(11)^. Two key observations support the scientific rationale for the immunomodulatory role of vitamin D. Firstly, the vitamin D receptor is expressed in cells of the innate and adaptive immune system, including B and T cells, monocytes, macrophages and dendritic cells. Secondly, immune cells express the enzyme 25(OH)D-1α-hydroxylase (CYP27B1) that acts to convert 25(OH)D_3_ to the active form, 1,25-dihydroxyvitamin D_3_ (1,25(OH)_2_D_3_)^(11)^. Cohort, case-control and cross-sectional studies in adults and children have demonstrated associations between low 25(OH)D concentrations and increased risk of ARTI^(12,13)^, but results from randomised controlled trials (RCT) of vitamin D supplementation have been less consistent^(14,15)^. A meta-analysis of 25 RCT including 11 321 participants found that vitamin D supplementation reduced the risk of ARTI by 11 %, and among those receiving daily or weekly vitamin D, protective effects were found to be stronger in those with vitamin D deficiency (<25 nmol/l) (OR: 0·30, 95 % CI: 0·17, 0·95)^(16)^. A more recent meta-analysis of 43 RCT including 48 488 participants found similar results, but it did not find enhanced protection in participants with the lowest 25(OH)D concentrations at baseline^(17)^. However, both meta-analyses reported considerable heterogeneity among trials.

Examining ethnic health disparities is crucial for setting public health priorities and maximising benefits for individuals. Of all published reviews on vitamin D and prevention of ARTI^(12,14,16–18)^, none have included a subgroup analysis for ethnicity. Additionally, no systematic review has summarised the evidence for the association between vitamin D and immune function in at-risk ethnic groups. Therefore, this systematic review aimed to examine the evidence for the relationship between vitamin D status or vitamin D supplementation in dark-skinned ethnic groups and the concomitant effects on both immune function biomarkers and prevention of ARTI. Additionally, a meta-analysis was performed to quantify the effect of vitamin D supplementation on ARTI incidence in ethnic subgroups.

## Methods

The review followed the Preferred Reporting Items for Systematic Reviews and Meta-Analyses guidelines^(19)^. A protocol had not been published *a priori*, nor was this review registered.

### Search strategy

Systematic literature searches were performed in six electronic databases (PubMed, Embase, Web of Science, ScienceDirect, Scopus and Cochrane Central) for published articles of all years of record up until the date of 15 December 2021. Search terms were determined by examining key words in the literature and were agreed by all authors. The search terms included ‘Vitamin D’ and ‘Immune Function’ and ‘Acute Respiratory Infection’ and ‘Ethnic Groups’. The complete search strategy can be found in the online supplementary material. Reference lists of included studies and relevant reviews were hand-searched for additional articles.

### Study eligibility criteria

Studies identified from the literature search were included when the following criteria were satisfied:

1. *Population:* As Fitzpatrick’s skin type is not always reported or measured within studies, participants from any age group belonging to an ethnic or racial group other than white, with or without a white comparator were included. If a white comparator was included, results were stratified by non-white ethnicity.
2. *Study design:* RCT (vitamin D supplementation *v*. placebo or other control), or cross-sectional, case-control, or cohort studies conducted in human subjects and reported in English language. *In vitro* or animal studies were excluded, along with review articles, book chapters, letters to the editor, conference papers, theses, case reports, abstract-only articles and meta-analyses.
3. *Exposure:* Examining the effect of serum concentrations of vitamin D metabolites, dietary intake of vitamin D (from food and/or supplementation) or vitamin D deficiency.
4. *Outcomes:* Biomarkers of immune function such as differential cell counts, concentrations of leucocyte types (e.g. leucocytes, lymphocytes, monocytes, neutrophils, granulocytes, dendritic cells), lymphocyte subsets (e.g. T, B, natural killer cells), antimicrobial proteins (e.g. cathelicidin, defensins), antibodies or antibody responses, immune cell activity, cytokines and C-reactive protein (CRP); incidence of ARTI, including upper respiratory tract infections and lower respiratory tract infections. Studies investigating COVID-19 were included, while tuberculosis and other chronic respiratory infections were excluded.

### Screening process

Retrieved articles were evaluated for relevancy using the software Rayyan (http://rayyan.qcri.org). Two reviewers (ARB and SALN) independently screened titles and abstracts of the identified articles. A third reviewer (KHH) provided clarification when required. Full-text articles were retrieved before excluding ethnic groups. Conflicts over included articles were resolved by consensus. Study authors of potentially eligible studies were contacted if clarification or more information was required.

### Data extraction

A customised data collection template was used to extract data from all studies. Data extracted included study characteristics (i.e. author, publication year, country), participant characteristics (i.e. population description), ethnic/racial groups (i.e. author-defined terms and collection method), age, sex, sample size, serum 25(OH)D concentrations and assay) and outcomes of interest (i.e. immune biomarkers, incidence frequency of ARI). If serum 25(OH)D was reported in ng/ml, it was converted to nmol/l by multiplying by 2·496 (1 ng/ml is equivalent to 2·496 nmol/l). For RCT, a description of the intervention and/or placebo and follow-up duration was also extracted. If vitamin D supplement dose was reported in international units, it was converted to µg (1 µg is equivalent to 40 international units). Statistical analyses and results were extracted for each outcome of interest, including number of participants (n) or events, mean, sd, correlation coefficients, *P* values and OR with 95 % CI. Where applicable, results were extracted from the models with the most covariates adjusted for.

### Quality assessment

The methodological quality evaluation of all studies was conducted by one researcher (ARB). The Jadad Scale assessment^(20)^ was used for RCT, the Newcastle–Ottawa Scale assessment^(21)^ was used for cohort studies, and a modified version of the Newcastle–Ottawa Scale by Herzog *et al.*^(22)^ was used for cross-sectional studies. The Jadad Scale awarded up to five points per study based on randomisation and method adequacy, blinding and method adequacy, as well as the accounting of all participants. The Newcastle–Ottawa Scale awarded up to nine stars for cohort studies while the adapted version awarded up to eight stars for cross-sectional studies for participant selection, comparability and assessment of outcome. A list of quality assessment criteria specific to the study subject was developed to standardise the decision for assessing risk of bias. Additionally, cut-off points were determined to aid the interpretation of total quality scores. Further details regarding these criteria and cut-offs can be found in the online supplement material (see online supplementary material, Supplementary Tables S1–3).

### Data synthesis

Results and statistical analyses of the included studies were presented in a tabular form, and a narrative synthesis was used to summarise the studies. A meta-analysis was conducted for studies reporting ARTI incidence in placebo-controlled trials with appropriate data and low risk of bias, and combined ethnic groups were described according to the most appropriate terms. Studies investigating immune biomarkers were not included in a quantitative meta-analysis as there was a large degree of heterogeneity in the methods for participant populations (e.g. disease characteristics, age categories), study design and outcomes measured. Moreover, of the three RCT investigating immune biomarkers, only two had usable data.

### Statistical analysis

The meta-analysis was performed using Review Manager (RevMan, Version 5·3, Copenhagen, The Nordic Cochrane Centre, The Cochrane Collaboration, 2014) software. Random-effects meta-analysis was conducted using the proportion of participants experiencing one or more ARTI in each ethnic group in the trials to obtain pooled OR and 95 % CI to estimate the effect of vitamin D supplementation on the risk of at least one ARTI compared to placebo. Heterogeneity was investigated using the *I*^2^ values of 25 %, 50 % and 75 % corresponding to low, moderate and high levels of heterogeneity, respectively. The number of studies was too small to perform a sensitivity analysis or to assess publication bias.

## Results

### Study selection

**Fig. 1:**
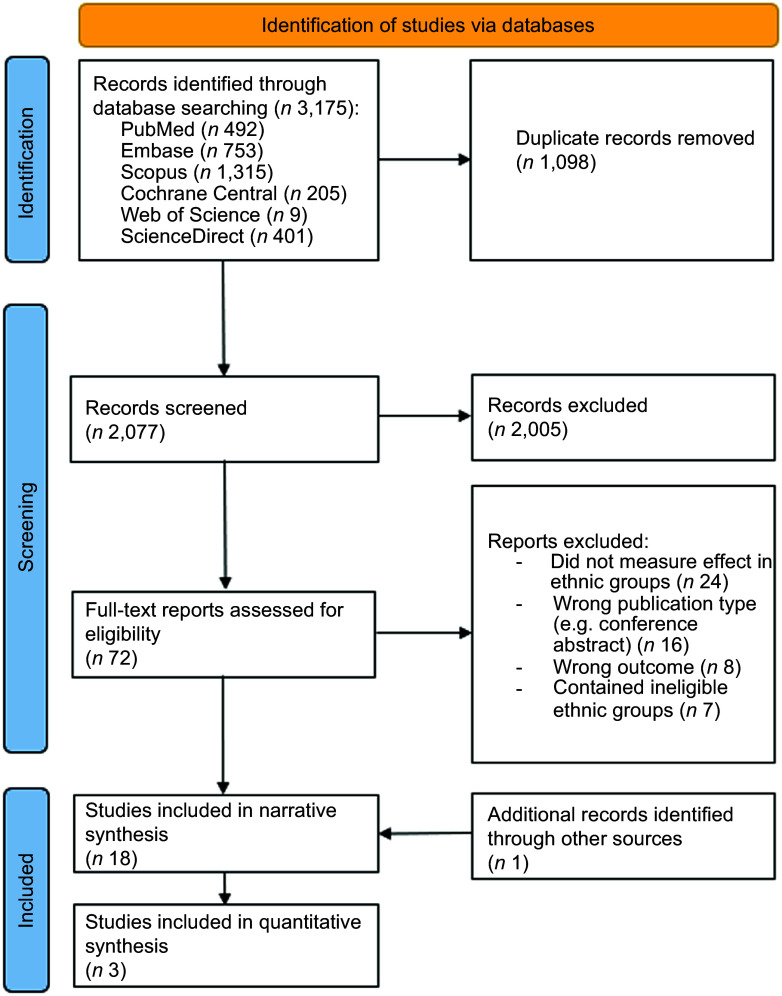
PRISMA flow chart of systematic review on the effect of vitamin D on immune function and prevention of acute respiratory infections

The details of the search strategy and selection process are presented in Fig. 1. The literature search identified 2077 articles for screening after duplicates were removed. After title and abstract screening, 2005 records were removed and full texts of 72 articles were assessed for inclusion. An additional article was identified from screened reference lists and included in the full-text screening. Of these studies, 18 articles fulfilled the eligibility criteria and were suitable for inclusion. Several articles were excluded after assessing full texts because ineligible ethnic groups were included in the analyses, or authors mentioned individuals were from a specific country but did not specify ethnicity or ethnic origin. Moreover, many studies were considered potentially eligible because ethnicity was mentioned in the baseline characteristics but were subsequently excluded because either no statistical analyses were reported for the effect in ethnic groups or ethnicity was only used as a covariate. Authors contacted did not respond to requests for data. A full breakdown of exclusion reasons is described in Fig. 1.

### Characteristics of included studies

Tables 1 and 2 present details of the characteristics of included studies. Seven RCT (39 %)^(23–29)^, seven cross-sectional studies (39 %)^(30–36)^ and four cohort studies (18 %)^(37–40)^ were included in the present review. The studies were published between 2013 to 2021 and were from the US (*n* 11; 61 %), India (*n* 1; 6 %); Malaysia (*n* 1; 6 %), Taiwan (*n* 1; 6 %), New Zealand (*n* 1; 6 %), Australia (*n* 1; 6 %), Canada (*n* 1; 6 %) and France (*n* 1; 6 %).

**Table 1:** Characteristics and outcomes of included RCT examining the effect of vitamin D on immune function or acute respiratory infections

				25(OH)D									
					Baseline nmol/l			Attained concentration in intervention group nmol/l					
Study & country	Population	Ethnicity/race; collection method	Vitamin D dose	Assay, EQA scheme	Mean	sd	Baseline <25 nmol/l, %	Mean	sd	Follow-up duration	Outcome	Main findings	Covariates adjusted for
Chandler *et al.* (2014), US^(23)^	328 healthy adults Age: 30–80 years	African American (100 %); Self-identified	25 µg, 50 µg or 100 µg daily or placebo	RIA (DiaSorin), EQA scheme NR	Median 25 µg: 40·4; Median 50 µg: 34·7; Median 100 µg: 39·2	IQR 25 µg: 26·0–58·9; IQR 50 µg: 23·7–55·7; IQR 100 µg: 27·5–58·2	NR	Median 25 µg: 74·1; Median 50 µg: 86·6; Median 100 µg: 114·6	IQR 25 µg: 63·9–82·1; IQR 50 µg: 71·9–137·3	3 months	IL-6 IL-10 sTNF-R2 CRP	No significant differences between treatment groups in concentrations of IL-6 (*P* = 0·84), IL-10 (*P* = 0·40), sTNF-R2 (*P* = 0·35) and CRP (*P* = 0·91). Baseline CRP was inversely associated with baseline 25(OH)D (*P* < 0·001)	–
Sinha-Hikim *et al.* (2015), US^(24)^	80 adults with pre-diabetes and hypovitaminosis D Age: ≥40 years	Latino (86 %), African American (14 %); NR	Adjusted weekly dose to maintain serum 25(OH)D 162–225 nmol/l or placebo	HPLC-MS/MS, EQA scheme NR	54·9	11·7	NR	174·7	NR	1 year	IL-6 TNF-α hs-CRP	No significant differences between treatment groups in concentrations of IL-6 (*P* = 0·67), TNF-α (*P* = 0·43) and hs-CRP (*P* = 0·43)	–
Varshney *et al.* (2019), India^(25)^	189 obese children and adolescents Age: 11–17 years	Asian Indian (100 %); NR	3000 µg bolus monthly or 300 µg monthly	CLIA (DiaSorin), DEQAS	20·9	13·6	70·6	67·1	30·5	12 months	IL6 TNFα hs-CRP	No significant differences between treatment groups (data not shown)	–
Aloia *et al.* (2019), US^(26)^	260 healthy older, female adults Age: >60 years	African American (100 %); Self-identified	Adjusted daily dose to maintain serum 25(OH)D > 75 nmol/l or placebo	LC-MS/MS, NIST	54·4	16·7	NR	117·3	28·0	3 years^*^	ARTI incidence	No significant difference in ARTI incidence over time between treatment groups (*P* = 0·775)	
Camargo *et al.* (2020), New Zealand^(27)^	5110 older adults Age: 50–84 years	European/Other (84 %), Māori (5 %), Pacific (6 %), South Asian (5 %); Self-identified	5000 µg bolus, then 2500 µg monthly or placebo	LC-MS/MS, DEQAS	63·0	24·0	2	135	40	3 years	ARTI incidence	No significant difference in ARTI incidence between treatment groups in Māori (*P* = 0·83), Pacific (*P* = 0·59) and South Asian (*P* = 0·19) subjects	
Denlinger *et al.* (2016), US^(28)^	408 adults with mild to moderate asthma Age: ≥18 years	white (53 %), African American (32 %), Hispanic (10 %), Asian/Pacific Islander (3 %), Other (2 %); NR	2500 µg bolus, then 100 µg daily or placebo	CLIA (DiaSorin), EQA scheme NR	46·9	(95 % CI: 45·4, 48·7)	13	104·6	95 % CI: 100·1, 109·1	28 weeks	URTI incidence	African American subjects receiving vitamin D compared to placebo had an increased rate of URTIs (*P* = 0·02)	
Hibbs *et al.* (2018), US^(29)^	300 preterm infants Age: Born 28 to 36 weeks’ gestation	black/African American (100 %); Parent identification	10 µg daily regardless of dietary intake or 10 µg daily if dietary intake was <5 µg, after which they received placebo	NR, EQA scheme NR	Median intervention: 46·7; Median control: 52·4	IQR intervention: 39·2–69·9; IQR control: 42·4–62·4	0	Median intervention: 82·4; Median control: 79·8	IQR intervention: 69·9–94·8; IQR control: 69·9–94·8	12 months	URTI and LRTI incidence	No significant difference in URTI or LRTI incidence between treatment groups (URTI difference, –1·6 %; 95 % CI: –17·1, 7·0; LRTI difference –3·6 %; 95 % CI: –16·4, 4·4)	

25(OH)D, 25 hydroxyvitamin D; ARTI, acute respiratory tract infection; CLIA, chemiluminescent immunoassay; CRP, C-reactive protein; DEQAS, vitamin D External Quality Assessment Scheme; EIA, enzyme immunoassay; EQA, external quality assessment; HR, hazard ratio; IQR, interquartile range; LC-MS/MS, liquid chromatography-tandem mass spectrometry; LRTI, lower respiratory tract infection; NIST, National Institute for Standards and Technology; NR, not reported; PBMC, peripheral blood mononuclear cell; RCT, randomised controlled trials; RR, risk ratio; TNF, tumour necrosis factor; US, United States; URTI, upper respiratory tract infection.

* Follow-up was 3 months for data contributing to meta-analysis.

**Table 2:** Characteristics and outcomes of included cross-sectional and cohort studies examining the effect of vitamin D on immune function and acute respiratory tract infections

				25(OH)D						
Study and country	Study design	Population	Ethnicity/race (%); collection method	Assay, EQA scheme	Mean nmol/l	sd nmol/l	<25 nmol/l, %	Outcome	Main findings	Covariates adjusted for
Antwi *et al.* (2018), US^(30)^	Cross-sectional	397 adults with T2D and without T2D Age: ≥35 years	Haitian American (50 %) African American (50 %); Self-identified	EIA, EQA scheme NR	Haitian American: 55·2 African American: 54·5	Haitian American: 18·9 African American: 24·4	NR	IL-6	Serum 25(OH)D was not associated with IL-6 (*P* > 0·05)	Age, energy intake, HOMA-IR
Bobbitt *et al.* (2015), US^(31)^	Cross-sectional	178 pregnant women Age: 18–44 years	African American (100 %); NR	CLIA, EQA scheme NR	33·4	21·0	NR	IL-6 IL-10 IL-1β TNF-α hs-CRP	Serum 25(OH)D was inversely associated with IL-1β (*P* = 0·027)	Age, pre-pregnancy BMI, Centre for Epidemiological Studies Depression Scale score, employment status, ≥high-school education, marital status, time between measures, season
Chen *et al.* (2018), US^(32)^	Cross-sectional	5681 participants from NHANES 2001 to 2004 Age: 6–49 years	Non-Hispanic white (46 %), Mexican American (26 %), Non-Hispanic black (19 %); Other Hispanic (5 %); Self-identified	RIA (DiaSorin), NIST	56·3	31·0	NR	Measles antibody titre	Serum 25(OH)D was not associated with measles antibody titre in Mexican American (*P* = 0·393) or non-Hispanic black (*P* = 0·083), Other Hispanic (*P* = 0·859), groups	Age, gender, alanine aminotransferase, total cholesterol, TAG, fasting glucose, calcium, creatinine, history of arthritis, congestive heart failure, CHD, angina/angina pectoris, heart attack, stroke, smoking, moderate to vigorous recreational activity
Gopal *et al.* (2019), Malaysia^(33)^	Cross-sectional	150 participants (100 rheumatoid arthritis patients; 50 controls) Age: ≤60 years	Malay (46 %), Chinese (29 %), Indian (25 %); NR	CLIA (Roche Cobas), EQA scheme NR	43·2	24·3	NR	IL-6 CRP	Baseline values and pre- and post-treatment with vitamin D was not associated with IL-6 (*P > 0·05*) or CRP (*P > 0·05*) in rheumatoid arthritis patients	–
Legeai *et al.* (2013), France^(34)^	Cross-sectional	355 adults with HIV Age: ≥18 years	white (57 %), black (43 %); NR	RIA (DiaSorin), EQA scheme NR	white: 46·7 black: 34·7	white: 25·5 black: 21·9	24	sTNF-R2 sTNF-R1 TNF-α IL-6 hs-CRP	In black subjects only, serum 25(OH)D < 25 nmol/l *v*. ≥25 nmol/l had significantly higher sTNF-R2 (*P* = 0·04) (data not shown for sTNF-R1, TNF-α, IL-6 and hs-CRP)	–
Szeto *et al.* (2020), US^(37)^	Cohort	93 adults hospitalised with COVID-19 Age: ≥18 years	black/African American (24 %), remaining NR; NR	CLIA, EQA scheme NR	Median: 62·4	(IQR: 42·4–82·4)	6	IL-6 CRP	In African American subjects, serum 25(OH)D < 50 nmol/l *v*. ≥50 nmol/l was not associated with IL-6 (*P* = 1·0) or CRP (*P* = 0·30)	–
Xiao *et al.* (2016), Canada^(35)^	Cross-sectional	514 adults Age: 20–79 years	white Canadian (54 %); South Asian (46 %); Self-identified	CLIA (DiaSorin), EQA scheme NR	white Canadian: 72·7 South Asian: 49·9	white Canadian: 32·4 South Asian: 24·2	NR	CRP	Serum 25(OH)D was not associated with CRP in South Asian subjects (*P* > 0·05).	Age, gender
Yao *et al.* (2014), Taiwan^(36)^	Cross-sectional	1315 children and adolescents Age: 5–18 years	Asian (100 %); Parent identification	ECLIA (Roche Elecsys), NIST	50·9	17·7	6·2	IgE	Serum 25(OH)D was not associated with IgE (*P* > 0·05)	Age, gender, BMI, season of sampling, passive smoking
Binks *et al.* (2016), Australia^(38)^	Nested Cohort	109 mother-infant pairs Gestation: 39 weeks	Indigenous (100 %); NR	ID-LC-MS, EQA scheme NR	54·0	21·0	10	LRTI hospitalisation in first year of infancy	Lower cord blood 25(OH)D was associated with increased infant LRTI hospitalisation (*P* = 0·025)	–
Crandell *et al.* (2021), US^(39)^	Retrospective cohort	16 602 adults with electronic health records Age: ≥18 years	white (82·9 %); black (17 %); NR	NR, EQA scheme NR	white: 88·1 black: 68·6 (39·7)	white: 42·2 black: 39·7	NR	SARS-CoV-2 positivity	Serum 25(OH)D was not associated with SARS-CoV-2 positivity in black individuals (*P* > 0·05)	–
Meltzer *et al.* (2021), US^(40)^	Retrospective cohort	4638 participants with electronic health records Mean age: 52·8 years	black (49 %); white (43 %), Other (8 %); Self-identified	NR, EQA scheme NR	75·6	39·7	NR	SARS-CoV-2 positivity	In black subjects only, serum 25(OH)D was inversely associated with SARS-CoV-2 positivity (*v*. ≥100 nmol/l: <50 nmol/l, *P* = 0·009; 75 to <100 nmol/l, *P* = 0·01) and positivity decreased by 5 % per 2·5-nmol/l increase in subjects with ≥75 nmol/l (30 ng/ml) (*P* = 0·03)	–

25(OH)D, 25 hydroxyvitamin D; ARTI, acute respiratory tract infection; CLIA, chemiluminescence immunoassay; CRP, C-reactive protein; EIA, enzyme immunoassay; ECLIA, electrochemiluminescence assay; EQA, external quality assessment; HIV, human immunodeficiency virus; HR, hazard ratio; hs-CRP, high sensitivity C-reactive protein; ID-LC-MS, isotope dilution-liquid chromatography-tandem mass spectrometry; IQR, interquartile range; LC-MS, liquid chromatography-tandem mass spectrometry; LRTI, lower respiratory tract infection; NHANES, National Health and Nutrition Examination Survey; NIST, National Institute for Standards and Technology; NR, not reported; PBMC, peripheral blood mononuclear cell; RCT, randomised controlled trials; RR, risk ratio; SARS-CoV, severe acute respiratory syndrome coronavirus; T2D, type 2 diabetes; TNF, tumour necrosis factor; sTNF-R, soluble tumour necrosis factor receptor; US, United States; URTI, upper respiratory tract infection.

Most participants were of white ethnicity (65 %), followed by black/African (23 %), Asian/Pacific (7 %), Mexican (2 %), Other (1 %), Māori (<1 %), Hispanic (<1 %) and Indigenous (<1 %) ethnicities. Nine articles reported the method for collecting data on ethnicity^(23,26,27,29,30,32,35,36,40)^. Self-identification was used in seven articles^(23,26,27,30,32,35,40)^, one used identification by parents^(29)^, and one identified children as Asian if born to parents of Asian descent^(36)^.

The review included a total of 36 707 (range: 80–16 602) participants. Participants’ age ranged from birth to 84 years and most studies were conducted in adults (>18 years) (*n* 11; 61 %)^(23,24,26–28,30,31,34,35,37,39)^. Seven studies reported the proportion of participants with vitamin D deficiency (25(OH)D < 25 nmol/l), which ranged from 2·0 % to 70·6 %^(25,27,28,34,36–38)^.

Four RCT compared the effects of a vitamin D regimen with placebo^(24,26–28)^, two compared higher *v*. lower dose vitamin D regimens^(25,29)^ and one compared the effects of three different doses of vitamin D regimens with placebo^(23)^. Immune function outcomes were reported in 11 studies^(23–25,30–37)^, while ARTI outcomes were reported in seven studies^(26–29,38–40)^.

### Assay methods

Fifteen studies provided information about the methods used to measure serum 25(OH)D^(23–28,30–38)^ and five studies standardised these measurements^(25–27,32,36)^. These methods included chemiluminescent immunoassay (*n* 6)^(25,28,31,33,35,37)^, RIA (*n* 3)^(23,32,34)^, liquid chromatography–tandem MS (LC-MS/MS; *n* 2)^(26,27)^, HPLC-MS/MS (*n* 1)^(24)^, electrochemiluminescence binding assay (*n* 1)^(36)^, enzyme immunoassay (*n* 1)^(30)^ and isotope dilution-liquid chromatography–tandem MS (ID-MS/MS; *n* 1)^(38)^. These measurements were standardised to the vitamin D External Quality Assessment Scheme (*n* 2) and National Institute of Standards and Technology (*n* 3).

### Risk of bias in studies

Table 3 shows the quality of evidence for the RCT^(23,24,26–29)^ using the Jadad Scale. Most trials reported an adequate method of randomisation (*n* 4)^(23,24,27,29)^. While blinding methods were mentioned in all trials, only three studies reported an adequate method of blinding^(23,24,27)^. An account of withdrawals and drop-outs was mentioned in most trials (*n* 6)^(23,25–29)^. Overall, the mean score was 4·14 out of 5, and all studies considered good quality.

**Table 3:** Quality appraisal of randomised controlled trials using Jadad scale^(20)^

Study	Randomisation (maximum 2 points)	Blinding (maximum 2 points)	Account of all participants (maximum 1 point)	Total score (out of 5)
Chandler *et al.* (2014)^(23)^	1	2	1	4
Sinha-Hikim (2015)^(24)^	1	2	0	3
Varshney *et al.* (2019)^(25)^	2	1	1	4
Aloia *et al.* (2019)^(26)^	2	1	1	4
Camargo *et al.* (2020)^(27)^	2	2	1	5
Denlinger *et al.* (2016)^(28)^	2	1	1	4
Hibbs *et al.* (2018)^(29)^	2	2	1	5

Tables 4 and 5 demonstrate the quality of evidence for the cross-sectional and cohort studies^(30–40)^ using the Newcastle–Ottawa Scale. Eight studies included a sample representative of the target population^(30–32,34–36,39,40)^ and seven studies adjusted for confounders^(30–32,35,36,39,40)^. Of the cross-sectional studies, five studies described non-respondents^(30,32,33,35,36)^, one study provided sample size justification^(32)^ and four reported an adequate measurement of association^(31–33,36)^. All studies described appropriate ascertainment of exposure and outcomes, and adequacy of follow-up was mentioned in all cohort studies. The mean score for cross-sectional studies was 5·57 out of 8, and for cohort studies, it was 7 stars out of 9, with most studies considered fair to good quality.

**Table 4:** Quality appraisal of cross-sectional studies using adapted Newcastle–Ottawa scale^(22)^

Study	Representativeness of the sample	Sample size	Non-respondents	Ascertainment of exposure	Comparability based on design and analysis	Assessment of outcome	Statistical test	Total (out of 8)
Antwi *et al.* (2018)^(30)^	*		*	*	**	*		**6**
Bobbitt *et al.* (2015)^(31)^	*			*	**	*	*	**6**
Chen *et al.* (2018)^(32)^	*	*	*	*	**	*	*	**8**
Gopal *et al.* (2018)^(33)^			*	*		*	*	**4**
Legeai *et al.* (2013)^(34)^	*			*		*		**3**
Xiao *et al.* (2016)^(35)^	*		*	*	*	*		**5**
Yao *et al.* (2014)^(36)^	*		*	*	**	*	*	**7**

**Table 5:** Quality appraisal of cohort studies using Newcastle–Ottawa scale^(21)^

Study	Representativeness of the sample	Selection of non-exposed	Outcome of interest does not present at start	Ascertainment of exposure	Comparability Based on design and analysis	Assessment of outcome	Follow-up long enough for outcomes to occur?	Adequacy of follow-up of cohorts	Total (out of 9)
Szeto *et al.* (2020)^(37)^		*	*	*		*	*	*	**6**
Binks *et al.* (2016)^(38)^		*	*	*		*	*	*	**6**
Crandell *et al.* (2021)^(39)^	*	*	*	*	*	*	*	*	**8**
Meltzer *et al.* (2021)^(40)^	*	*	*	*	*	*	*	*	**8**

### Effect of vitamin D on biomarkers of immune function

#### Inflammation

Of the nine studies reporting on biomarkers of inflammation (three RCT; five cross-sectional studies; and one cohort study), most were performed in adults^(23,24,30,31,34,35,37)^, including one study in pregnant women^(31)^. Six studies focused on subjects with inflammatory conditions^(24,25,30,33)^ or acute/chronic infections^(34,37)^. Average baseline 25(OH) status in RCT ranged from 20·9 to 54·9 nmol/l. Among these trials, two did not report the prevalence of vitamin D deficiency (<25 nmol/l) at baseline^(23,24)^, while one reported a prevalence of 70·6 %^(25)^. The vitamin D_3_ dosing regimens varied, ranging from 25 to 100 µg daily for three months^(23)^, average weekly doses (mean ± sd) of 2133 µg ± 400 for 1 year^(24)^ or monthly doses of 3000 µg or 300 µg for 12 months^(25)^. The attained 25(OH)D concentrations following these interventions are described in Table 1. The main biomarkers measured were CRP (*n* 8)^(23–25,31,33–35,37)^ and IL-6 (*n* 8)^(23–25,30,31,33,34,37)^. Three studies found a significant association between vitamin D status and at least one inflammatory biomarker in black populations^(23,31,34)^. No significant change was found in IL-6, IL-10, TNF alpha (TNF-α), CRP or soluble TNF receptor 2 (sTNF-R2) monitored within an RCT following vitamin D supplementation compared to placebo or control in African American, Latino or Asian Indian ethnic groups^(23–25)^.

Chandler *et al.* found a significant inverse association between baseline 25(OH)D status and CRP concentrations in African American adults, but no significant changes were found following vitamin D_3_ supplementation of 25 µg, 50 µg or 100 µg daily^(23)^. In the remaining two RCT measuring CRP, no significant difference between treatment groups was found following average weekly doses (mean ± sd) of 2133 µg ± 400 in Latino and African American adults with pre-diabetes and hypovitaminosis D^(24)^ or monthly doses of 3000 µg or 300 µg in Asian Indian obese children and adolescents^(25)^. There were no significant findings after adjusting for covariates in four observational studies measuring CRP concentrations in American pregnant women^(31)^, Asian participants with rheumatoid arthritis^(33)^, black adults with HIV^(34)^ and black adults hospitalised with COVID-19^(37)^.

Bobbitt *et al.* found a significant inverse association between 25(OH)D and IL-1β concentrations in African American pregnant women after adjusting for covariates^(31)^. No significant difference between treatment groups was found in three RCT measuring IL concentrations (IL-6 or IL-10) following 25 µg, 50 µg or 100 µg daily in African American adults^(23)^, average weekly doses (mean ± sd) of 2133 µg ± 400 in Latino and African American adults with pre-diabetes and hypovitaminosis D^(24)^ or monthly doses of 3000 µg or 300 µg in Asian Indian obese children and adolescents^(25)^. There were no significant findings after adjusting for covariates in four observational studies measuring IL concentrations (IL-6 or IL-10) in non-Hispanic black type 2 diabetic and non-diabetic adults^(30)^, Asian participants with rheumatoid arthritis^(33)^, black adults with HIV^(34)^ and black adults hospitalised with COVID-19^(37)^.

Five studies investigated TNF-α (*n* 2), sTNF-R2 (*n* 2) or soluble TNF receptor 1 (sTNF-R1) (*n* 1))^(23–25,31,34)^. Legeai *et al.* found black adults with HIV and vitamin D deficiency (<25 nmol/l) had significantly higher sTNF-R2 concentrations compared to those who were not deficient^(34)^. No significant difference between treatment groups was found for these biomarkers in three RCT measuring IL concentrations (IL-6 or IL-10) following 25 µg, 50 µg or 100 µg daily in African American adults^(23)^, average weekly doses (mean ± sd) of 2133 µg ± 400 in Latino and African American adults with pre-diabetes and hypovitaminosis D^(24)^ or monthly doses of 3000 µg or 300 µg in Asian Indian obese children and adolescents^(25)^. Bobbit *et al.* found no significant association between 25(OH)D concentrations and TNF-α in African American pregnant women^(31)^.

#### Antibody concentrations

Two cross-sectional studies investigated antibody concentrations^(32,36)^, yet no significant association with 25(OH)D was found for IgE concentrations in Asian children and adolescents^(36)^ or measles antibody titres in Mexican American, non-Hispanic black or Other Hispanic participants after adjusting for covariates^(32)^.

### Effect of vitamin D on acute respiratory tract infection incidence

Seven studies reported on incidence of ARTI (four RCT; three cohort studies)^(26–29,38–40)^. Among the RCT, most were performed in adults (*n* 3)^(26–28)^, while one study was performed in infants^(29)^. Average baseline 25(OH)D status of RCT ranged from 46·9 to 63·0 nmol/l. The prevalence of vitamin D deficiency (<25 nmol/l) at baseline was reported in three out of four RCT, ranging from 0 to 13 %^(26–29)^. The vitamin D_3_ dosing regimens included bolus doses of 2500 µg then 100 µg daily for 28 weeks^(28)^ or 5000 µg then 2500 µg monthly for 3 years^(27)^, an adjusted daily dose (mean ± sd) of 87·3 µg ± 36·6 for 3 years^(26)^ or 10 µg daily regardless of dietary intake^(29)^. The attained 25(OH)D concentrations following these interventions are described in Table 1. RCT investigated ARTI incidence, and cohort studies measured COVID-19 incidence or hospitalisation due to lower respiratory tract infections.

Most RCT^(26,27,29)^ did not find any significant difference between treatment groups. However, Denlinger *et al.* found a significantly higher incidence of ARTI in the placebo group compared to the intervention group in African American adults with asthma, but no significant difference between treatment groups was found in Hispanic, Asian/Pacific Islander or Other ethnic groups^(28)^. Of the two cohort studies investigating COVID-19 incidence in black and white individuals, only one found a significant inverse association between serum 25(OH)D and SARS-CoV-2 positivity in black individuals^(40)^. Binks *et al.* in Indigenous mother-infant pairs found that lower mean cord blood 25(OH)D concentrations were associated with lower respiratory tract infection hospitalisation in infants compared to those that were not hospitalised^(38)^.

### Meta-analysis for the effect of vitamin D3 supplementation on acute respiratory tract infection incidence

**Fig. 2:**
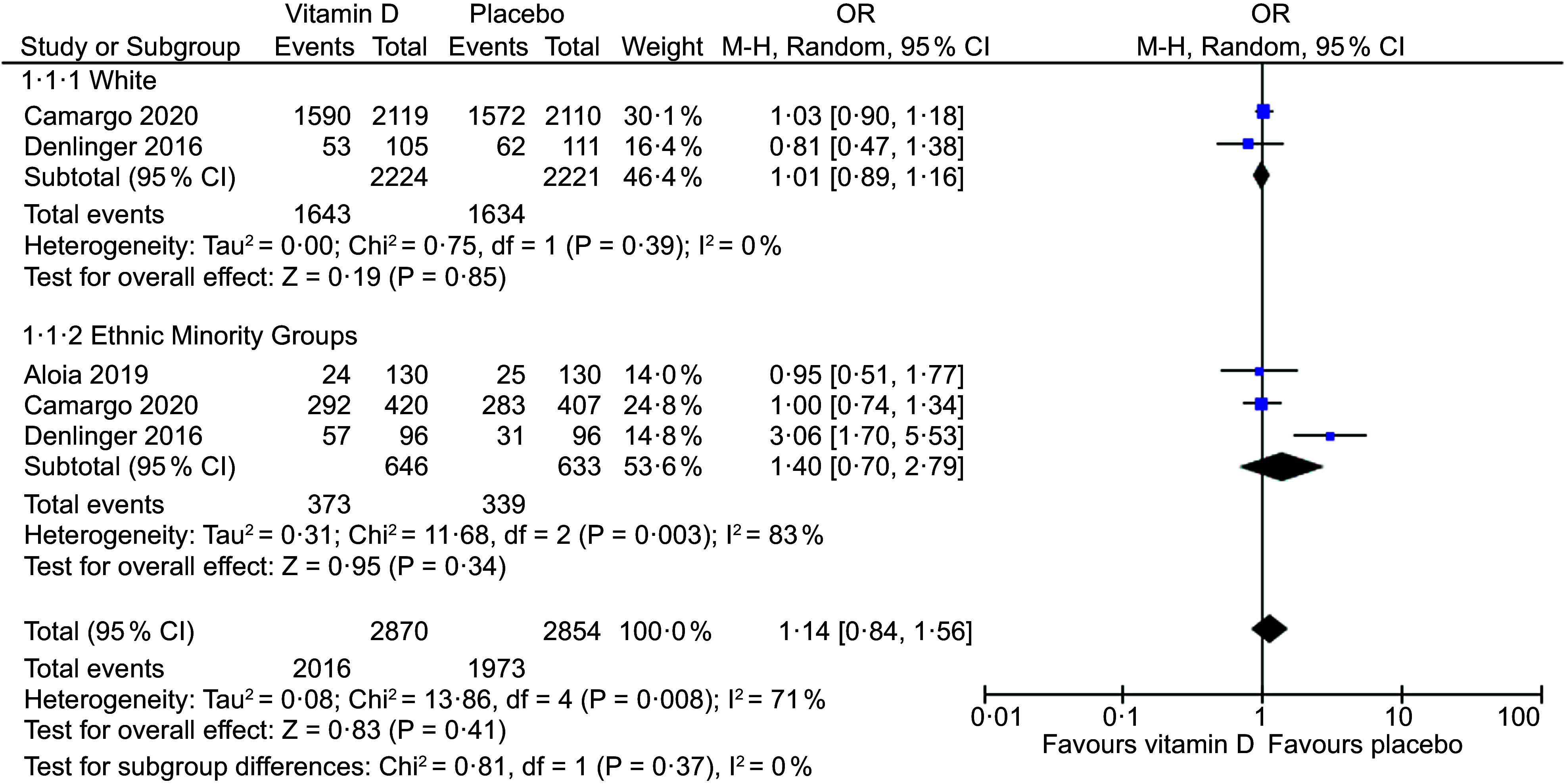
Forest plot of placebo-controlled trials investigating the effect of vitamin D supplementation on acute respiratory tract infection (ARTI) incidence in adults, sub-grouped by white and ethnic minority groups. Individual trial effect estimates (boxes) and pooled effect estimate (diamond) for ARTI incidence are shown. Values are OR with error bars representing the 95 % CI determined with the use of M-H random-effects models. Heterogeneity was quantified by *I*^2^ at a significance of *P* < 0·10

**Fig. 3:**
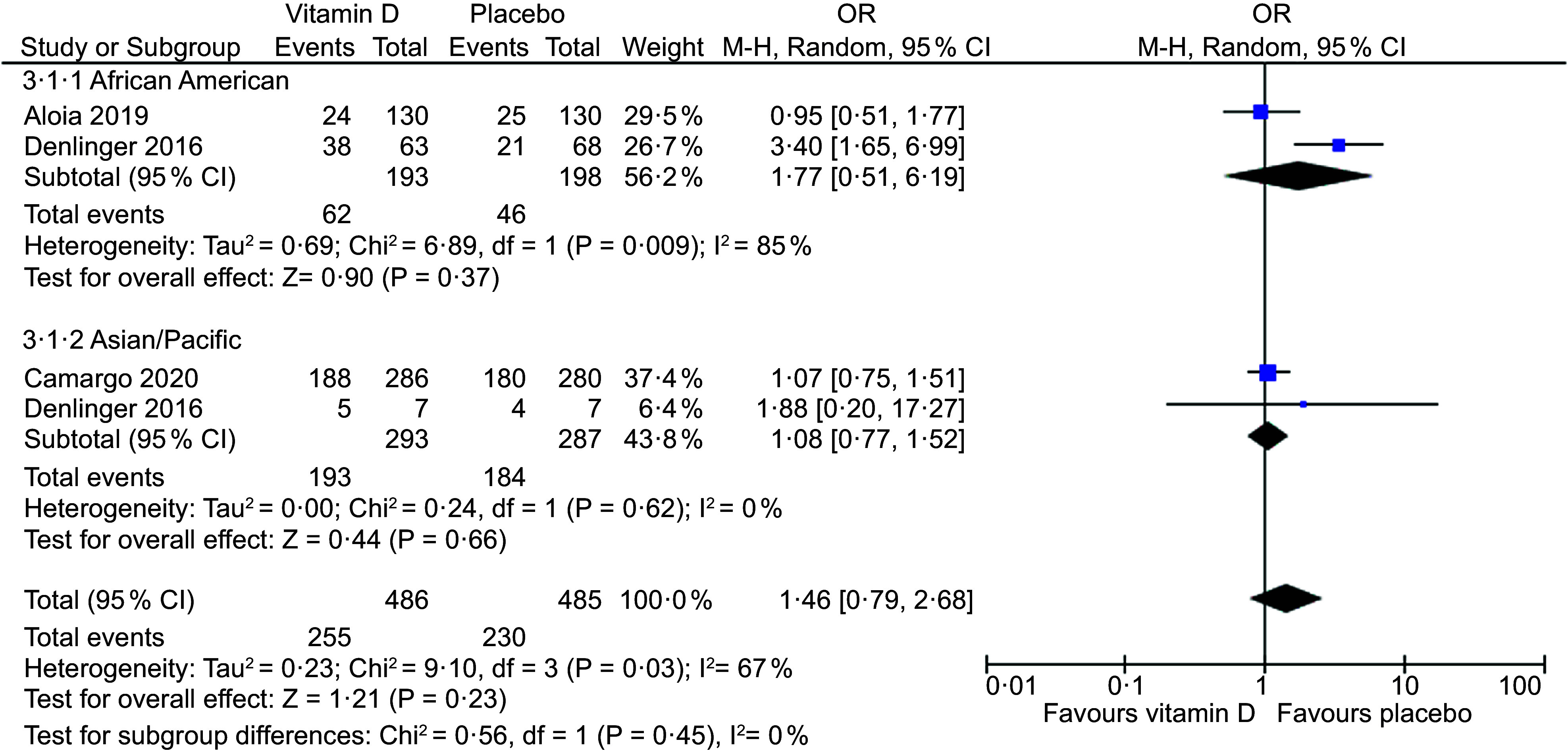
Forest plot of placebo-controlled trials investigating the effect of vitamin D supplementation on acute respiratory tract infection (ARTI) incidence in adults, sub-grouped by African American and Asian/Pacific ethnic groups. Individual trial effect estimates (boxes) and pooled effect estimate (diamond) for ARTI incidence are shown. Values in the plot are OR with 95 % CI determined with the use of M-H random-effects models. Heterogeneity was quantified by *I*^2^ at a significance of *P* < 0·10

Three placebo-controlled RCT in adults provided sufficient data to be included in the meta-analysis^(26–28)^. There was no statistically significant difference in ARTI incidence between vitamin D supplementation and placebo groups overall (OR, 1·14; 95 % CI: 0·84, 1·56; *P* = 0·41) and in the subgroup analysis for ethnic minority groups (South Asian, Pacific, Asian/Pacific Islander, Māori, American Indian/Alaska Native, African American, Hispanic, Other) (OR, 1·40; 95 % CI: 0·70, 2·79; *P* = 0·34) (Fig. 2). In the subgroup analysis, there was high heterogeneity in the ethnic minority group (*I*^2^ = 83 %). A second subgroup analysis was performed in African American and Asian/Pacific (South Asian, Pacific, Asian/Pacific Islander) groups (*n* 2). No statistically significant differences were found overall (OR, 1·46; 95 % CI: 0·79, 2·68; *P* = 0·23) and in African American (OR, 1·77; 95 % CI: 0·51, 6·19; *P* = 0·37) or Asian/Pacific (OR, 1·08; 95 % CI: 0·77, 2·68; *P* = 0·66) groups (Fig. 3). In this subgroup analysis, heterogeneity was higher in the African American group (*I*^2^ = 85 %).

## Discussion

This systematic review investigated whether vitamin D status or vitamin D supplementation is associated with biomarkers of immune function and prevention of ARTI in dark-skinned ethnic groups with diverse subject characteristics and geographical settings. While some cohort and cross-sectional studies found vitamin D status to be inversely associated with inflammation and ARTI, the results were conflicting. No evidence was identified to support the use of vitamin D supplementation to reduce inflammation or ARTI. However, the existing literature addressing this issue is limited.

Previous human-derived immune cell studies have shown that vitamin D has many effects on cells within the immune system, including the production of antimicrobial peptides and cytokines, regulating of nuclear factor-kB and reducing the activation of genes encoding inflammatory factors^(41)^. In this review, significant inverse associations with vitamin D status were identified for CRP^(23)^, sTNF-R2^(34)^ and IL-1β^(31)^ in black/African American individuals. However, no significant association was found in studies investigating the relationship between vitamin D status and IL-6^(23,30,31,33,34,37)^, IL-10^(23,31)^, sTNF-R1^(34)^, TNF-α^(31,34)^, IgE^(36)^ or measles antibody tires^(32)^ in dark-skinned ethnic groups. The subject populations that showed significant results had average serum 25(OH)D concentrations <40 nmol/l^(23,31,34)^, indicating that conflicting results may be due to threshold effects. Nonetheless, there were no significant findings for the effect of vitamin D supplementation on concentrations of IL-6, TNF-α, CRP^(23–25)^, IL-10 or sTNF-R2^(23)^ in dark-skinned ethnic groups. These results are consistent with a systematic review that found no significant effect on CRP and TNF-α in overweight and obese adults^(42)^. However, while previous meta-analyses of RCT investigating vitamin D supplementation on inflammatory biomarkers have reported inconsistent findings^(43–45)^, some have shown significant effects for at least one biomarker of inflammation, including IL-6, TNF-α or CRP, in patients with heart failure^(43)^, type 2 diabetes^(45)^ and abnormal glucose homeostasis^(44)^.

The interaction between adiposity and immunomodulatory or inflammatory mediators may influence the differential response to vitamin D_3_. For instance, in the vitamin D and Omega-3 Trial, vitamin D supplementation was linked to lower advanced cancer rates in participants, with the strongest reduction found in individuals with normal BMI and no reduction in individuals who were overweight or obese^(46)^. Additionally, it has been found that the 25(OH)D increase in the intervention arm and population heterogeneity might impact the power of vitamin D RCT investigating immunomodulatory mediators^(47)^ or respiratory infections^(48)^. Moreover, large increases in 25(OH)D concentrations might not achieve sufficient power if the population is already 25(OH)D sufficient^(48)^. In this review, only one RCT investigated a predominantly vitamin D deficient population (70·6 %) at baseline^(25)^. Following high-dose vitamin D supplementation, only 41 % of subjects in the intervention group achieved adequate vitamin D concentrations (>75 nmol/l) and 32 % remained vitamin D insufficient (<50 nmol/l). Therefore, sufficient concentrations of 25(OH)D may not have been achieved to identify significant effects on inflammatory biomarkers, highlighting a need for further well-designed RCT.

The preventative role of vitamin D against both bacterial and viral ARTI has been proposed due to its antibacterial and antiviral properties^(41)^. In this review, two cohort studies identified an inverse association between vitamin D status and ARTI incidence in black^(40)^ and Indigenous groups^(38)^. However, conflicting results were found in studies investigating the association between vitamin D status and COVID-19 positivity in black and white individuals^(39,40)^. These studies used 25(OH)D measurements at 90 d and 1 year before the COVID-19 test, which could account for the conflicting findings as vitamin D status can change over time^(39)^.

The evidence presented in the meta-analysis of RCT did not show a statistically significant difference when comparing vitamin D supplementation and a placebo among ethnic minorities, or African American or Asian/Pacific subgroups. The null results in the current study should be interpreted with caution due to the low number of studies included in the meta-analysis. In contrast, several meta-analyses in adults, children or adolescents reported a significant reduction in ARTI with vitamin D supplementation compared to the control^(12,16–18,49)^. Contradictory reports to this review may be attributed to differences in subject populations, as previous studies did not investigate ethnic groups. Additionally, there may be possible differences in ARTI pathogens, as lower respiratory tract infections and upper respiratory tract infections are associated with different aetiologies^(50)^.

There did not appear to be any clear trends in the quality scores and the significance of the findings. However, while scoring criteria were developed for the Jadad and Newcastle–Ottawa Scales to minimise bias from the subjective interpretation of scoring, these assessment methods were not appropriately designed for all studies and the quality scores were therefore used as a general guide to assess quality.

### Strengths and limitations

This review has several strengths, such as the broad range of outcomes examined to provide a comprehensive overview of the relationship between vitamin D and both immune function and ARTI. As this subject area is an emerging area of research, the search terms and inclusion criteria were extensive to capture all relevant clinical studies.

Limitations to this review include the low number of studies together with considerable heterogeneity between studies for ethnic groups studied, study design, quality and method for analysing 25(OH)D concentrations. The accuracy of vitamin D status may be reduced as only two studies reported using LC-MS/MS to measure 25(OH)D, which is considered the gold standard^(51)^. The health status varied between studies, with several populations having pre-existing medical conditions that may impact immune function or ARTI.

The method for classifying ethnicity was not mentioned in 50 % of included studies, potentially leading to inaccuracies in reporting. However, of the eight studies that did report the collection method, most used self-reported ethnic identification which is generally considered the ideal method of collection^(52)^. Moreover, it is recognised that combining ethnic minorities into one group in a meta-analysis is a limitation as ethnic group differences may be overlooked. While a second analysis was conducted, not all individual ethnic groups were able to be combined due to country-specific differences in ethnic classification and/or author reporting, limiting the comparability of certain ethnic groups.

The definition of vitamin D deficiency used in the present study is <25 nmol/l, as recommended by the Scientific Advisory Committee on Nutrition^(2)^, whereas the Institute of Medicine and European Food Safety Authority use < 30 nmol/l to indicate an increased risk of vitamin D deficiency^(1,53)^. The lack of consensus on these thresholds may be a reason for the low number of studies that provided data on vitamin D deficiency. Of the studies that did provide data, there was a lack of studies investigating populations with low baseline 25(OH)D status. Another possible limitation may be the heterogeneity in dosing regimens adopted by the studies. Daily dosing regimens using standard doses (10–25 µg) taken for up to 12 months have been found to provide the most benefit against ARTI^(17)^, yet most RCT investigating ARTI in this review used large daily^(26)^ or monthly doses^(27,28)^.

### Future research

Drawing accurate conclusions is challenging due to the small number of studies found, with only seven RCT included in this review. The limited number of studies compared to prior reviews on vitamin D supplementation and ARTI that did not consider ethnic groups^(17,18)^ highlights the importance of improved reporting of ethnicity, and the need for further research in this area, particularly RCT. Despite many studies included in this review having large sample sizes, 65 % of all subjects were of white ethnicity. This highlights the underrepresentation of dark-skinned populations, despite their higher risk of vitamin D deficiency^(3,4)^ and ARTI-related adverse outcomes^(6,54)^. To increase the participation rate when targeting ethnic groups, it has been suggested that recruitment strategies should be culturally sensitive, including community outreach and researchers who are members of the same ethnic group^(55)^. Therefore, improved guidelines on collecting and reporting ethnicity in future research are needed.

None of the studies included in this review investigated the association between vitamin D and differential cell counts, leucocyte types, lymphocyte subsets or antimicrobial proteins in dark-skinned populations, highlighting a gap in the literature. Previous research conducted *in vitro* and *in vivo* has demonstrated that vitamin D can alter many cells in the immune system^(41)^. A study by Liu *et al.*^(56)^ reported that African American subjects had significantly lower vitamin D_3_ compared to Caucasians, and induction of cathelicidin mRNA was significantly reduced in the presence of serum from African American subjects. This evidence suggests that adequate vitamin D concentrations are required for cathelicidin production. Hence, future trials investigating a range of immune biomarkers in dark-skinned populations are warranted.

## Conclusions

This review found a lack of conclusive evidence supporting an association between vitamin D status and immune function or ARTI incidence in dark-skinned ethnic groups. No evidence was found to support the use of vitamin D supplementation in reducing inflammation or ARTI within these populations. The findings are limited by the small number of RCT with considerable heterogeneity between studies for baseline 25(OH)D status and vitamin D dosing regimens and a lack of studies investigating low vitamin D status. Hence, further RCT investigating dark-skinned populations with low vitamin D status and using appropriate vitamin D dosing regimens are needed to elucidate the link between vitamin D health, immune function and ARTI.

## Supporting information

Bournot et al. supplementary material Bournot et al. supplementary material

## Supplementary material

For supplementary material accompanying this paper visit https://doi.org/10.1017/S1368980024001861
