# Supplementary material for: Effect of vitamin D status and vitamin D supplementation on immune function and prevention of acute respiratory tract infections in dark-skinned individuals: a systematic review and meta-analysis
Source: Public Health Nutr. 2024 Oct 28;27(1):e224. doi: 10.1017/S1368980024001861 (PMC11645122; doi:10.1017/S1368980024001861)
Supplement: Bournot et al. supplementary material [file S1368980024001861sup001.docx]

# **Supplement****ary Material**

**Effect of vitamin D status and vitamin D supplementation on immune function and prevention of acute respiratory tract infections in dark-skinned individuals: a systematic review and meta-analysis**

This file contains:

- Supplementary Text for electronic searches
- Quality assessment criteria (Tables S.1-3)

**Supplementary Text**

An advanced search was performed in all databases for all years in Dec 2021.

1. **PubMed**

All fields searched: (“vitamin D” OR “vitamin D2” OR “vitamin D3” OR cholecalciferol OR ergocalciferol OR alphacalcidol OR alfacalcidol OR calcitriol OR paricalcitol OR doxercalciferol OR 25(OH)D OR 25-hydroxy*) AND (“immune function” OR immunity OR immun* OR “immune system” OR “innate immunity” OR “humoral immunity” OR “adaptive immunity” OR “cellular immunity” OR “immune markers” OR cytokines OR chemokines OR leukocytes OR neutrophils OR lymphocytes OR “T lymphocytes” OR “T cells” OR “T-cells” OR “B lymphocytes” OR “B cells” OR “B-cells” OR “T-regs” OR “T regs” OR “T regulatory cells” OR “T-regulatory cells” OR monocytes OR macrophages OR “dendritic cells” OR “natural killer cells” OR interferons OR interleukins OR “tumour necrosis factor” OR “acute phase protein” OR “antimicrobial peptides” OR granulocytes OR “acute respiratory infection” OR “upper respiratory infection” OR “lower respiratory infection” OR “respiratory tract infection”) AND (“ethnic group” OR race OR ethnicity OR migrant OR black OR Asian OR “Afro-Caribbean”)

1. **Embase**

Title, abstract, or keywords searched: ('vitamin d' OR 'vitamin d2' OR 'vitamin d3' OR cholecalciferol OR ergocalciferol OR alphacalcidol OR alfacalcidol OR calcitriol OR paricalcitol OR doxercalciferol OR 25ohd OR '25 hydroxy*') AND ('immune function' OR immunity OR immun* OR 'immune system' OR 'innate immunity' OR 'humoral immunity' OR 'adaptive immunity' OR 'cellular immunity' OR 'immune markers' OR cytokines OR chemokines OR leukocytes OR neutrophils OR lymphocytes OR 't lymphocytes' OR 't cells' OR 'b lymphocytes' OR 'b cells' OR 't regs' OR 't-regs' OR 't-regulatory cells' OR 't regulatory cells' OR monocytes OR macrophages OR 'dendritic cells' OR 'natural killer cells' OR interferons OR interleukins OR 'tumour necrosis factor' OR 'acute phase protein' OR 'antimicrobial peptides' OR granulocytes OR ‘acute respiratory infection’ OR ‘upper respiratory infection’ OR ‘lower respiratory infection’ OR ‘respiratory tract infection’) AND ('ethnic group' OR race OR ethnicity OR migrant OR black OR asian OR 'afro caribbean')

1. **Scopus**

TITLE-ABS-KEY((“vitamin D” OR “vitamin D2” OR “vitamin D3” OR cholecalciferol OR ergocalciferol OR alphacalcidol OR alfacalcidol OR calcitriol OR paricalcitol OR doxercalciferol OR 25OHD OR 25-hydroxy*) AND (“immune function” OR immunity OR immun* OR “immune system” OR “innate immunity” OR “humoral immunity” OR “adaptive immunity” OR “cellular immunity” OR “immune markers” OR cytokines OR chemokines OR leukocytes OR neutrophils OR lymphocytes OR “T lymphocytes” OR “T cells” OR “T-cells” OR “B lymphocytes” OR “B cells” OR “B-cells” OR “T-regs” OR “T regs” OR “T regulatory cells” OR “T-regulatory cells” OR monocytes OR macrophages OR “dendritic cells” OR “natural killer cells” OR interferons OR interleukins OR “tumour necrosis factor” OR “acute phase protein” OR “antimicrobial peptides” OR granulocytes OR “acute respiratory infection” OR “upper respiratory infection” OR “lower respiratory infection” OR “respiratory tract infection”) AND (“ethnic group” OR race OR ethnicity OR migrant OR black OR Asian OR “Afro-Caribbean”)

1. **Cochrane Central**

Title, abstract, or keywords searched: ('vitamin d' OR 'vitamin d2' OR 'vitamin d3' OR cholecalciferol OR ergocalciferol OR alphacalcidol OR alfacalcidol OR calcitriol OR paricalcitol OR doxercalciferol OR 25ohd OR '25 hydroxy*') AND ('immune function' OR immunity OR immun* OR 'immune system' OR 'innate immunity' OR 'humoral immunity' OR 'adaptive immunity' OR 'cellular immunity' OR 'immune markers' OR cytokines OR chemokines OR leukocytes OR neutrophils OR lymphocytes OR 't lymphocytes' OR 't cells' OR 'b lymphocytes' OR 'b cells' OR 't regs' OR 't-regs' OR 't-regulatory cells' OR 't regulatory cells' OR monocytes OR macrophages OR 'dendritic cells' OR 'natural killer cells' OR interferons OR interleukins OR 'tumour necrosis factor' OR 'acute phase protein' OR 'antimicrobial peptides' OR granulocytes OR ‘acute respiratory infection’ OR ‘upper respiratory infection’ OR ‘lower respiratory infection’ OR ‘respiratory tract infection’) AND ('ethnic group' OR race OR ethnicity OR migrant OR black OR asian OR 'afro caribbean')

1. **ScienceDirect**

Title, abstract, or keywords searched: ("vitamin D") AND ("immune function" OR "immune system" OR "immune markers" OR "acute respiratory tract infection") AND (ethnicity OR "ethnic group")

1. **Web of Science**

TS=(("vitamin D" OR "vitamin D2" OR "vitamin D3" OR cholecalciferol OR ergocalciferol OR alphacalcidol OR alfacalcidol OR calcitriol OR paricalcitol OR doxercalciferol OR 25(OH)D OR 25-hydroxy*) AND ("immune function" OR immunity OR immun* OR "immune system" OR "innate immunity" OR "humoral immunity" OR "adaptive immunity" OR "cellular immunity" OR "immune markers" OR "acute respiratory infection" OR "upper respiratory infection" OR "lower respiratory infection" OR "respiratory tract infection") AND ("ethnic group" OR race OR ethnicity OR migrant OR black OR Asian OR "Afro-Caribbean")

**Table S.1**. Scoring criteria for the Jadad Scale to assess quality of studies (out of a total of 5 stars)

| **Criteria** | **Point awarded** | **Point not awarded** |
| --- | --- | --- |
| Randomisation (2 points) | Randomisation mentioned and second point for adequate method (e.g., computed generated random number) | No description or inappropriate method described |
| Blinding (2 points) | Blinding mentioned and second point for adequate method (e.g., identical placebo tablets) | No description or inappropriate method described |
| Account of all participants (1 point) | Adequate description of all participants including withdrawals and drop-outs | No description |

Thresholds for converting the Jadad Scale:

The study was considered as good quality for the total Jadad score of ≥3, otherwise it was considered as poor quality.

**Table S.2**. Scoring criteria for the Newcastle-Ottawa Scale (adapted version) to assess quality of cross-sectional studies (out of a total of 8 stars)

| **Category** | **Criteria** | **Star awarded** | **Star not awarded** |
| --- | --- | --- | --- |
| **Selection** | Representativeness of the sample (1 star) | Respondent sampling was used to recruit participants or the study reanalysed data from another study | Clinic sample |
|  | Sample size (1 star) | Justified and satisfactory | Not justified |
|  | Non-respondents (1 star) | Characteristics of non-respondents and respondents is established, and response rate is satisfactory. Alternatively, response rate is 100% | Response rate is unsatisfactory or no description |
|  | Ascertainment of exposure (1 star) | Medical records or directly measured using a laboratory test | No description |
| **Comparability** | Comparability (2 stars) | The study controlled for the principal factors that were relevant to the outcomes measured (e.g., age, sex); second star awarded if the study controlled for additional demographic characteristics or comorbidities | No description |
| **Outcome** | Ascertainment of outcome (1 star) | Medical records or directly measured using a laboratory test | No description |
|  | Statistical test (1 star) | The statistical test was clearly described and appropriate, with confidence intervals and *P* values presented | Statistical test is not appropriate or not described |

Note: In contrast to the criteria developed by Herzog et al. (2013), this review assigned one star for ascertainment of exposure and outcome in cross-sectional studies for adequately describing the laboratory test or medical records.

Thresholds for converting the adapted Newcastle-Ottawa Scale (good, fair, and poor):

**Good quality**: 3 or 4 stars in selection domain AND 1 or 2 stars in compatibility domain AND 1 or 2 stars in outcome/exposure domain

**Fair quality**: 2 stars in selection domain AND 1 or 2 stars in comparability domain AND 1 or 2 stars in outcome/exposure domain

**Poor quality**: 0 or 1 star in selection domain OR 0 stars in comparability domain OR 0 stars in outcome/exposure domain.

**Table S.3**. Scoring criteria for the Newcastle-Ottawa Scale to assess quality of cohort studies (out of a total of 9 stars)

| **Category** | **Criteria** | **Star awarded** | **Star not awarded** |
| --- | --- | --- | --- |
| **Selection** | Representativeness of the sample (1 star) | Population-based | Clinic sample |
|  | Selection of non-exposed cohort (1 star) | Same population setting for all ethnic groups | Different population setting or no description |
|  | Ascertainment of exposure (1 star) | Medical records or directly measured using a laboratory test | No description |
|  | Demonstration that outcome of interest was not present at start of study (1 star) | Adequate description of study population and outcome of interest at start of study | No description |
| **Comparability** | Comparability (2 stars) | The study controlled for factors that were relevant to the outcomes measured (e.g., age, sex); second star awarded if the study controlled for additional demographic characteristics or comorbidities | No description |
| **Outcome** | Ascertainment of outcome (1 star) | Medical records or directly measured using a laboratory test | No description |
|  | Follow-up long enough for outcomes to occur (1 star) | Follow-up duration adequate | No description or follow-up duration inadequate |
|  | Adequacy of follow-up of cohorts (1 star) | No missing outcome data or reasons for missing outcome data are unlikely to be related to true outcome | Missing outcome data with reasons likely to be related to true outcome |

Thresholds for converting the Newcastle-Ottawa Scale (good, fair, and poor):

**Good quality:** 3 or 4 stars in selection domain AND 1 or 2 stars in comparability domain AND 2 or 3 stars in outcome/exposure domain

**Fair quality:** 2 stars in selection domain AND 1 or 2 stars in comparability domain AND 2 or 3 stars in outcome/exposure domain

**Poor quality:** 0 or 1 star in selection domain OR 0 stars in comparability domain OR 0 or 1 stars in outcome/exposure domain

**Supplementary Material Reference**

1. Herzog R, Álvarez-Pasquin MJ, Díaz C, del Barrio JL, Estrada JM, Gil Á. Are healthcare workers intentions to vaccinate related to their knowledge, beliefs and attitudes? A systematic review. BMC Public Health. 2013;13(1).
